# Study of the Pozzolanic Reactivity of Sugar Cane Straw Ashes (SCSA) Burned under Controlled Conditions

**DOI:** 10.3390/ma16216841

**Published:** 2023-10-24

**Authors:** Michelle S. Rodrigues, Jordi Payá, Lourdes Soriano, José Monzó, María Victoria Borrachero, Holmer Savastano, Antonio L. Beraldo

**Affiliations:** 1 School of Agricultural Engineering—FEAGRI, State University of Campinas, Campinas 13083-896, SP, Brazil; michatcsr@gmail.com (M.S.R.); beraldo@feagri.unicamp.br (A.L.B.); 2 Giquima—Research Group of Building Materials Chemistry, Instituto de Ciencia y Tecnología del Hormigón ICITECH, Universitat Politècnica de València, 46022 Valencia, Spain; lousomar@upvnet.upv.es (L.S.); jmmonzo@cst.upv.es (J.M.); vborrachero@cst.upv.es (M.V.B.); 3 Department of Biosystem Engineering, University of São Paulo, Pirassununga 13635-900, SP, Brazil; holmersj@usp.br

**Keywords:** biomass ash, sugar cane waste, paste, pozzolan, compressive strength

## Abstract

The aims of this work were to evaluate the reactivity of sugarcane straw ashes (SCSA) burned under controlled conditions and to analyze their reactivity in blended cement and hydrated lime pastes by thermogravimetric analysis (TG) and calorimetry. Four different ashes were produced, and burned at 600 °C, 700 °C, 800 °C and 900 °C (SCSA600, SCSA700, SCSA800 and SCSA900, respectively). These ashes were characterized by X-ray fluorescence spectroscopy, X-ray diffractometry, particle size distribution by laser diffraction and specific area surfaces to assess their potential interest in the partial replacement of inorganic binders (Portland cement (OPC) and hydrated lime). The hydrated lime pastes were subjected to scanning electron microscopy (SEM) and TG. The blended cement pastes were analyzed by TG and calorimetry, compressive strength testing and mercury intrusion porosimetry. High lime fixation percentages were observed in the hydrated lime and OPC pastes and were higher than 75% and 50% for the ashes burned at 600 °C and 700 °C, respectively. Calorimetry showed a delay in the heat release of SCSA600 and SCSA700 compared to the control paste. These pastes also had higher compressive strength and a smaller total pore volume. The results indicate the positive response of preparing sugar cane ashes under controlled conditions (mainly for straw calcined within the 600–700 °C range) for their use as pozzolanic addition by partially replacing inorganic binders.

## 1. Introduction

Around 7–8% of global CO_2_ emissions of anthropogenic origin are due to cement production [1]. Approximately 798 kg of CO_2_ are emitted per ton of clinker and are produced mainly by raw materials such as limestone and fuel [1]. Of the alternatives for reducing cement industry emissions, several have been proposed: using alternative fuels, improving manufacturing processes, employing alternative clinker types (belitic and calcium sulfoaluminate clinker) or utilizing supplementary cementitious materials [2].

Agriculture generates 19.9% of CO_2_ emissions, second to the energy sector (68.1%) and produces a large amount of waste [3]. Some of this waste could mitigate the impact on CO_2_ emissions if biomass is used as an energy source. The conversion of this biomass into energy would save around 50 billion tons of oil. The valorization of biomass as a source of energy is being increasingly implemented worldwide. However, biomass conversion produces an additional waste—biomass ash [4].

The use of ash derived from the combustion of agro-industrial waste as supplementary cementitious materials (SCM) has been studied in several research works in the last few decades [5,6]. There is a diversity of agro-industrial waste, such as rice husk ash (RHA), wood ash (WA), rice straw ash (RSA), palm oil fuel ash (POFA) and corn cob ash (CCA), among others [6]. They all share the common feature of containing SiO_2_, Al_2_O_3_, K_2_O and Fe_2_O_3_ as the main components in their composition, although the main oxide is CaO in WA [7]. RHA is still the most studied waste for being employed as pozzolan [8,9,10,11]. Hassan et al. [9] studied substituting up to 20% of cement for RHA to fabricate concrete. They demonstrated that better compressive strength was obtained by the concrete with 10% RHA (68.92 MPa at 28 curing days). Although it is noteworthy that concrete with 20% RHA obtained 61.5 MPa at 28 curing days compared to 65 MPa for the control system. These findings are very positive because very similar values are obtained even when replacing 20% of cement in concrete. However, one negative characteristic of this ash is a loss of workability in the concrete containing RHA. 

The temperature at which biomass combustion takes place has a great influence on the characteristics of the ash obtained. Thus, Xu et al. [12] analyzed the reactivity of RHA obtained at 500, 600, 700 and 800 °C. It was observed that a crystallization of amorphous silica to cristobalite occurred at 800 °C, which reduced the reactivity of the ash in mixtures with Portland cement.

Sugar cane production in Brazil has considerably increased and was approximately 607 million tons in 2022/2023 [13]. With mechanical sugarcane harvesting, another by-product is generated: sugar cane straw (composed of leaves and stems), which can be used in animal feeding, pulp, papermaking and as biomass. 

Many studies have reported the use of sugarcane bagasse ash (SCBA), which has a very high potential to be applied as a mineral addition. Research works studying the use of SCBA as pozzolan have concluded that, due to the rough surface and irregular shape of particles, the workability of mixtures with this pozzolan is poorer than that of the corresponding control mixtures [14,15,16]. As for the optimal percentage of cement substitution for SCBA to obtain good mechanical strengths, the authors place this percentage within the 10–20% range [17,18,19].

The use of sugar cane straw ash (SCSA) has been less investigated than SCBA, but several publications show good pozzolanic behavior [20,21,22]. One of the first references is that published by Villar-Cociña et al. [20], where its pozzolanic effect was demonstrated by taking electrical conductivity measurements in solutions saturated with lime. Moraes et al. [23] used SCSA, which they obtained by autocombustion. They concluded that this pozzolan offered an important pozzolanic contribution in mortars by replacing percentages of Portland cement (PC) by up to 30%. Pozzolanic activity was observed after only three curing days. 

The hydration process of ordinary Portland cement (OPC) with mineral additions is considered very complex because it involves reactions of mineral additions or pozzolanic materials, in addition to the hydration of OPC itself. Thermogravimetric (TG) and calorimetric analyses can be used to analyze the hydration of the pastes containing these materials [24]. The calorimetry method can provide continuous measurements and proves to be a good method for studying the initial phase when the hydration heat rate is relatively high. This technique is widely used by researchers to assess the reactivity of different pozzolans. However, it is difficult to monitor the pozzolanic reaction with this technique because the pozzolanic reaction may occur at older ages and it has a low heat rate [25,26]. 

The TG technique is suitable for studying hydration in blended pastes [25,27]. Moropoulou et al. [28] investigated the pozzolanic activity of pastes with lime and pozzolan by TG. The authors concluded that fixed lime, as determined by this technique in pastes, may be an indicative factor for assessing the reactivity of pozzolans, and long-term curing ages (i.e., 90 and 180 days) should be investigated.

The aims of this study were to evaluate the reactivity of SCSA burned under different conditions by means of physico-chemical characterizations and to study their reactivity in inorganic binder-based pastes by calorimetry and TG. There are few studies on the analysis of the reactivity of SCSA obtained at different combustion temperatures, and the results can offer ways for the industrial valorization of this waste in the field of construction materials based on Portland cement.

## 2. Materials and Methods

### 2.1. Materials

The sugar cane straw used in this work was collected from sugarcane plants in São Paulo State, Brazil. SCSA samples were produced in a laboratory furnace at 600 °C, 700 °C, 800 °C and 900 °C (SCSA600, SCSA700, SCSA800 and SCSA900, respectively). First, straw was calcined at 400 °C for 20 min and then burned at the corresponding temperature for 60 min. The heating rate was 10 °C·min^−1^, and cooling was natural. SCSA grinding was carried out in a laboratory ball mill for 120 min [29]. 

Brazilian type V cement (high early strength Portland cement (95% clinker in its composition)) of high initial strength was employed according to Brazilian standard ABNT NBR 16697 [30,31]. This cement (acronym used OPC) was used for preparing OPC control pastes and SCSA-containing pastes, and it was equivalent cement type III ASTM [32] and to cement type CEM I (European Committee for Standardization, CEN) [33]. Calcium hydroxide (hydrated lime, CH) of 95% purity was used to prepare CH/SCSA pastes. Deionized water was used for the experimental program.

### 2.2. Ashes Characterization

Chemical composition was obtained by X-ray fluorescence spectroscopy with an X Axios Panalytical apparatus. Loss on ignition (LOI) was determined by heating the sample up to 950 ± 50 °C according to ASTM C-114 [32]. 

The mineralogical characterization of ashes was carried out by the X-ray diffraction (XRD) test to evaluate the analyzed material’s structure. X-ray patterns were obtained with a 6-circle Huber diffractometer, a Ge (111) analyzing crystal and a Mythens detector at 9 keV (λ 3776). Data were obtained at room temperature using a θ–2θ geometry with a flat support for the sample in a 2θ range of 10°–70° with a step of 0.01° and a time of 1 s. The MATCH 1.1 software was used to identify the phases in the ashes.

The granulometric distributions of cement and ashes were measured using a Malvern Mastersizer 2000 apparatus, which allows the analysis of particle size by laser diffraction from 0.02 to 2000 μm in the liquid mode as a dispersant, with 10–15% obscuration and ultrasonic agitation for 60 s. The specific surface area of ashes was determined using the BET technique (Brunauer-Emmett-Teller), obtained by nitrogen absorption.

### 2.3. Mixing Procedures for Pastes

In order to study the hydration process of cement with ashes, two different kinds of mixtures were prepared: OPC- and calcium hydroxide-(CH)-based pastes. They appear in Table 1.

The water/binder (w/b) ratios for all the mixtures appear in Table 1, according to the previous literature [34,35,36,37]. After mixing materials, according to the procedures of Brazilian Standards ABNT NBR 7215 [38], pastes were stored in sealed plastic bottles and then left in a curing room at 20 °C for the testing ages of 7, 28, 63 and 91 days. The blended CH-based pastes were evaluated by TG and SEM. The OPC-based pastes (control and blended) were submitted to TG, heat of hydration analysis, compressive strength testing and porosimetry analysis. 

### 2.4. Thermogravimetric Analysis (TG) 

The TG was carried out to evaluate the fixed lime in the cement and hydrated lime pastes [34]. Mixtures were tested at 7, 28, 63 and 91 days. Small samples were extracted from these pastes, ground in an agate mortar and mixed with acetone to stop the hydration process. These samples were then filtered and dried at 60 °C for 1 h. The employed equipment was a Mettler-Toledo TG 850 with a balance (0.1 µg resolution). The heating method went from 35 to 600 °C at a heating rate of 10 °C.min^−1^ in a 100 µL aluminum crucible with a pinhole in the sealing lid for a self-generated atmosphere under an inert atmosphere of dry N2 flow (75 mL.min^−1^).

Pozzolanic activity was evaluated by the determination of the fixed lime in both paste types. The fixed lime in the cement pastes (CH_cp_) was calculated using the data obtained in the control paste with only cement and the paste with SCSA and cement according to Equations (1) and (2) [39]:

(1) $$Fixed\;{CH}_{cp}(\%)=\frac{\left[\left({CH}_{c}\right)\;\cdot {\;C}_{\%}\right]-{\left(CH\right)}_{SCSA-cp}}{\left[\left({CH}_{c}\right)\;\cdot \;\;C_{\%}\right]}.100$$

(2) $$CH=\frac{H}{PM_{H}}\cdot PM_{CH}$$

where CH_c_ = CH is the amount in the paste with only cement; (CH)_SCSA-cp_ = CH is the amount in the paste with SCSA; C% = relative cement content in the paste (0.8 in this case because replacement was 20%); H = water mass loss within the 520–580 °C range; PM_H_ = molecular mass of water; PM_CH_ = molecular mass of calcium hydroxide.

In the pastes with hydrated lime, the fixed lime (CH_hlp_) was calculated by Equation (3) [39]:

(3) $$Fixed\;{CH}_{hlp}(\%)=\frac{{\left(CH\right)}_{o}-{\left(CH\right)}_{SCSA-hlp}}{{\left(CH\right)}_{o}}\cdot 100$$

where CH_o_ = CH initial amount in the paste; (CH)_SCSA-hlp_ = CH final amount in the paste.

### 2.5. Scanning Electron Microscopy (SEM) of the CH-Based Pastes

The pastes with CH and ash at 91 curing days were analyzed by SEM. A JEOL JSM6300 was employed by applying the 20 kV voltage to the sample. The X-ray analysis software (AZtec 3.3 SP1 Oxford Instruments) for energy dispersive spectroscopy (EDS) acquisition, quantification and image acquisition was employed. 

### 2.6. Hydration Heat of the Blended Pastes and OPC Paste

The study of the hydration heat of the pastes was conducted to determine the influence of ashes in the early hydration process stages [40]. The evaluated mixtures were the pastes described in Table 1. Pastes were prepared by mixing ash with OPC (20:80 mass ratio) and deionized water (w:b mass ratio 0.5). Pastes were placed inside glass ampoules and connected to the channels in the isothermal calorimeter (TA Instruments, Eden Prairie, MN, USA; TAM air). Pastes were monitored for a 72 h period. The sample mass was variable for each test. Therefore, the results were normalized so that a comparative analysis could be carried out between the control sample and those with ashes (mass of binder). To normalize, the signal obtained by the equipment was divided by the mass of the sample. To this end, the curves were expressed as heat flux (W/g of binder) versus time (h). For the accumulated heat curve (J/g of binder), the initial minutes were not disregarded with all data included on the curve.

### 2.7. Compressive Strength Testing of the Blended and Control Cement Pastes

In order to carry out the compression strength testing and porosimetry of pastes, 23 cylindrical specimens (25 mm diameter, 50 mm high) were produced per mixture. Twenty-two of them were used in the mechanical test (28 and 91 days) and one sample in the mercury intrusion porosimetry test (age of 91 curing days). 

The universal testing machine EMIC was used, model DL-30000, equipped with a 50 kN load cell. The actuator displacement speed was 0.3 mm.min^−1^. To calculate compressive strength, the rupture load was divided by the specimen’s section area to obtain the average of the 11 samples for each curing age. Data were analyzed using the statistical software Statgraphics Plus 5.1 for Windows, Centurion version, and were submitted to a multifactor analysis of variance (ANOVA) to evaluate the effect of the produced paste types (control, OPC/SCSA600, OPC/SCSA700, OPC/SCSA800 and OPC/SCSA900). Means were compared by Tukey’s test at a 5% statistical significance level.

### 2.8. Mercury Intrusion Porosimetry (MIP) of the Blended and Control Cement Pastes

The MIP analysis was carried out for the pastes at a curing age of 91 days. This technique provides the pore size distribution of the sample with the amount of mercury introduced into the material according to the intrusion pressure and the corresponding pore size. The employed equipment was a Micromeritics mercury porosimeter, model Poresizer 9320. The cubic sample of each paste, with 10 mm of the edge, was extracted from the central region of the minicylindrical specimens (25 mm diameter, 50 mm high) using a precision cutter (Struers brand, Miniton model with an M1D15 cutting disc). 

## 3. Results

### 3.1. Physico-Chemical Characterization of Ashes

Table 2 shows the chemical composition and LOI of ashes. The sum of oxides SiO_2_, Al_2_O_3_ and Fe_2_O_3_ was more than 70% in all the SCSA samples (range 77.2–79.8%). According to ASTM C618 [41], these materials would have a potential composition to be considered pozzolanic admixtures. SiO_2_ content was higher than 60% and obtained a value higher than that reported previously, such as that reported by Moraes et al. [23], which was 36.5%. This value was slightly lower than the value indicated by other such as Frías et al. [42], which was 70%. Undesirable oxides, such as K_2_O and SO_3_, were lower than predicted by standards, as was LOI. 

**Figure 1:**
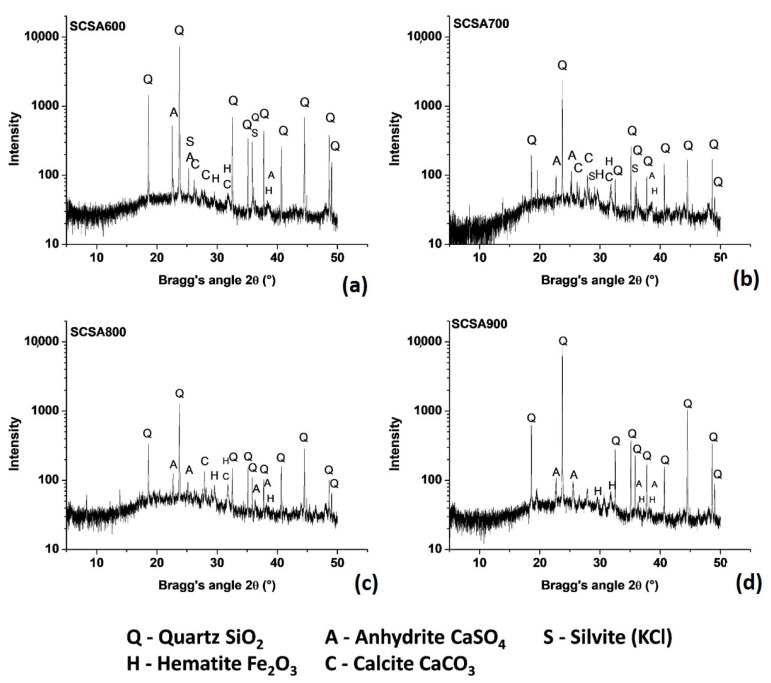
XRD patterns of ashes: (**a**) SCSA600, (**b**) SCSA700, (**c**) SCSA800 and (**d**) SCSA900.

Figure 1 depicts the XRD patterns for ashes (SCSA). All the samples presented crystalline phases, which can be attributed to the impurities present in straw; these impurities are related to soil composition. Sample SCSA600 displayed the presence of quartz (Q), hematite (H) and calcite (C), the typical minerals present in the soil where sugarcane was grown. Additionally, the presence of anhydrite (A) and silvite (K) was related to the minerals present in sugarcane leaves and stems. These minerals were also present in the SCSA700. However, in SCSA800, silvite was absent because of its evaporation (sublimation) at this temperature. For SCSA900, calcite had also disappeared because of the decarbonation process. For all the samples, a halo of amorphicity was recorded as the baseline deviation reflected with a Bragg angle (2θ) between 15° and 30°. This halo amorphicity is indicative of the potential reactivity of the evaluated ashes. No cristobalite and trydimite formation was observed for the sample treated at the highest temperature, which indicated that amorphous silica was stable under the studied conditions, and the low cooling rate had no consequence on the mineralogy of silica. Frías et al. [42] found that cristobalite formed for the SCSA calcined at 1000 °C, although the halo in the XRD pattern did not disappear.

**Figure 2:**
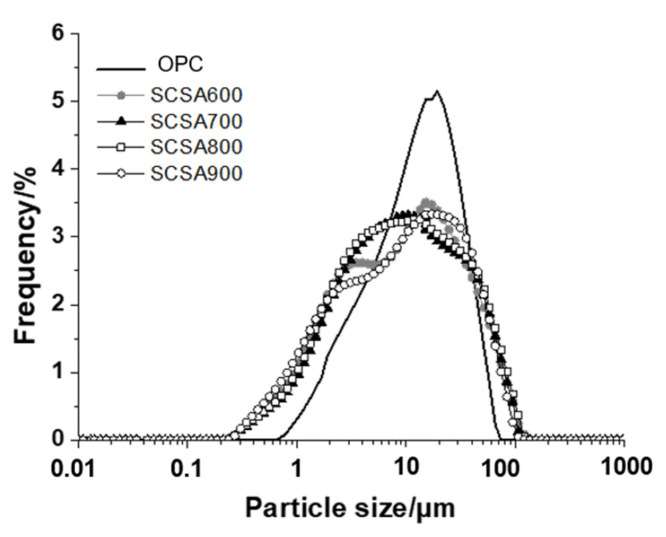
Particle size distribution curves for the cement (OPC) and SCSA samples.

After grinding, all the samples presented a similar particle size distribution. The particle size versus frequency of the samples are presented in Figure 2. Samples had an average diameter close to 17 µm and 90% volume of particles were below 45 µm. Thus, they all presented marked fineness.

The BET analysis showed that ashes SCSA600, SCSA700, SCSA800 and SCSA900 had specific surface areas of 33.9 m^2^·g^−1^, 12.2 m^2^·g^−1^, 8.03 m^2^·g^−1^ and 5.33 m^2^·g^−1^, respectively. These values revealed that as the burning temperature increased, the specific surface area of ashes decreased. The effects of both the heating rate and temperature make the crystallization/synterization process vary. Hence the variations in the BET values [43]. SCSA800 and SCSA900 obtained low specific surface area values, possibly due to the structure of the sintered particles promoted by burning, which changed the volume of the micropores present in the particles. Therefore, the SCSA600 ash was the most porous of them all. This behavior was observed by Cordeiro and Sales [44]: elephant grass ash (EGA) was obtained in the burning range of 500 to 900 °C. The BET value was reduced from 45.6 to 20.3 m^2^/g. Xiong et al. [45] proposed that porous silica obtained at low burning temperatures has many silanol (Si-OH) groups. As the burning temperature increases, the adjacent silanol groups react, and a condensation reaction takes place. This process collapses the porous structure. In addition, these authors proposed an additional mechanism that reduces the specific surface area: a solid-state reaction promoted by the presence of potassium and phosphorous, yielding crystallization/sinterization reactions. The SCSA600 had a high specific surface area, denoting high internal porosity, whereas the SCSA700, SCSA800 and SCSA900 had a smaller specific surface area due to the sintered structure of the particles, smooth surface and low porosity due to the high temperatures and the residence in the furnace for a longer period during natural cooling, which changed the volume of micropores present in the particles.

### 3.2. Hydration Heat of Pastes

**Figure 3:**
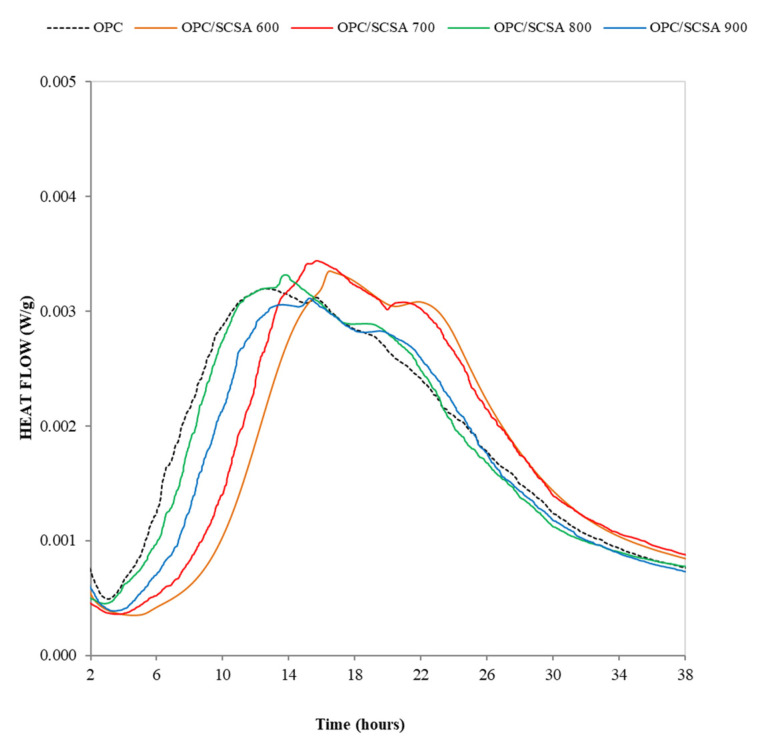
The hydration heat curves (in W per g of binder) of pastes during the 2–38-h period.

Figure 3 shows the heat flow rate versus time (from 2 to 38 h) curves that reflect the hydration process of the control and blended cement pastes. The typical first peak associated with the former hydrolysis of the OPC components [46] is not represented in Figure 3. The curve began 2 h after the induction (or dormant) period. The first peak represented in Figure 3 corresponds to C_3_S hydration and normally appears after a 9 h reaction. In parallel, during C*_3_*S hydration, ettringite precipitated continuously, and a part of sulfate was adsorbed on C-S-H. When sulfate was depleted from the system, the second peak occurred, which corresponds to more ettringite formation and to the reaction of C_3_A [47,48]. In the present research, the first peak associated with C_3_S in the control paste occurred around 13 h, with the second peak at about 16 h. When ash was added, peaks were delayed in relation to the control, which appeared at 17, 16, 14 and 15 h for the pastes with ashes SCSA600, SCSA700, SCSA800 and SCSA900, respectively. The second peak was delayed in relation to the control and appeared at 22, 21, 19 and 20 h, respectively, for the same ashes. As shown, in this case, the presence of SCSA delayed the appearance of the hydration peaks. Normally, the presence of SCM promotes the displacement of the peak of the C*_3_*S reaction to earlier ages due to action at nucleation points by the SCM material, which improves C*_3_*S hydration [49]. The data obtained in the present research are consistent with those obtained by de Siquiera and Cordeiro [40], who found delays in peaks appearing during calorimetry when they employed SCBA. Those authors highlighted that their data were consistent with those obtained when studying setting times, where the presence of SCBA led to a delay in the initial and final setting times. The explanation for this phenomenon can be found in this material’s greater fineness, which translates into water absorption and, therefore, a delay in setting processes. 

The SCSA600 ash was the most porous of the ashes. For this reason, perhaps this ash absorbed more water and delayed the hydration process in the first reaction steps. Additionally, the presence of unburned material also delays the hydration reaction, as has been reported by Ruviaro et al. [50]. These authors showed a delay in the maximum heat flux when oat husk ash was used as a pozzolanic material; increasing the calcination temperature of the oat husk meant a reduction in this delay effect on cement hydration. In relation to the second peak (aluminate), the sulfate depletion process is observed to occur after the main peak (silicate), which is attributed to the system being correctly dosed in terms of sulfate (supersulfated) [50].

**Figure 4:**
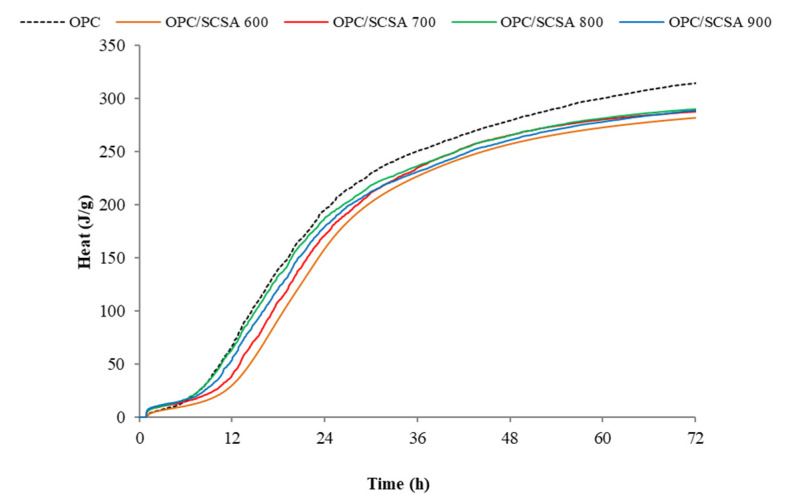
Accumulated heat curves of pastes (in J per g of binder).

Figure 4 shows the accumulated heat curves of pastes. After 72 h of analysis, it was verified that the pastes with SCSA600, SCSA700, SCSA800 and SCSA900, respectively, presented 89.9%, 91.4%, 92.2% and 91.8% accumulated heat in relation to the value obtained for the control. This means that despite containing 20% less cement, these pastes obtained values that came very close to those of the control paste. The heat released during hydration in the pastes with SCSA was derived from cement hydration and also from the pozzolanic and nucleation effects of SCSA, despite the delay observed in peaks appearing. 

### 3.3. Thermogravimetric and Microscopic Analyses of the Hydrated Lime and OPC Pastes 

The percentage of fixed lime in the pastes was analyzed in the blended cement pastes and CH pastes. Table 3 the shows fixed lime (CH_hlp_) evolution by age for the CH/SCSA pastes. 

Pastes showed high fixed lime levels even at 7 days, except for CH/SCSA900 with only 50% fixed CH, which was expected considering the smallest specific surface area and the highest degree of crystallization. Paste CH/SCSA600 displayed the highest hydrated lime consumption compared to the other pastes. This fixed lime remained similar for the 7–91-day period (an increase in the material’s surface area enhances the chemical reaction rate). This means that the pozzolanic reaction for SCSA600 occurred in the first 7 days. When comparing these results to the others consulted in the literature, lime fixation was higher in this study, and there was even more lime than in the study by Payá et al. [39], who obtained lime fixations of 43% using SCBA in pastes with a CH/SCBA ratio of 3/7. 

The lime fixation results are lower than those reported by Moraes et al. [23], who obtained 100% lime fixations after 3 curing days. This value is higher, but the CH/SCSA ratio was 3/7, and hence, there was less lime to fix.

**Figure 5:**
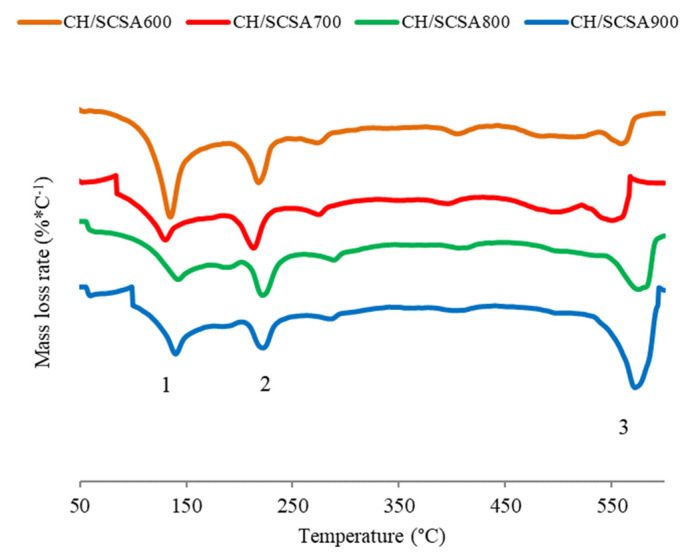
DTG curves for the CH/SCSA pastes at the curing age of 63 days.

Figure 5 shows the differential thermogravimetric (DTG) curves for the pastes with CH and SCSA after 63 curing days, when the fixed lime values began to stabilize and the maximum CH consumption was reached. On those curves, the peak between 100 °C and 180 °C (zone 1) was attributed to calcium silicate hydrate (CSH) dehydration; the peak between 180 °C and 250 °C (zone 2) was related to the decomposition of both calcium silicoaluminate hydrate (CASH) and calcium aluminate hydrate (CAH); the peak between 520 °C and 580 °C (zone 3) referred to CH dehydroxylation [39]. Thus, CSH dehydration was more pronounced for the CH/SCSA600 paste. The decomposition of both CASH and CAH was similar for all the pastes. As for the peak of the mass loss corresponding to CH decomposition (releasing water within the 520–580 °C range), the CH/SCSA900 paste had the highest mass loss of all the pastes, which indicates lower CH consumption by ash. CH/SCSA600 showed the least water loss from Ca(OH)*_2_* dehydroxylation. This was due to CH consumption and calcium silicate/aluminate hydrate formation, as confirmed by the increases in the CSH, CASH and CAH peaks.

**Figure 6:**
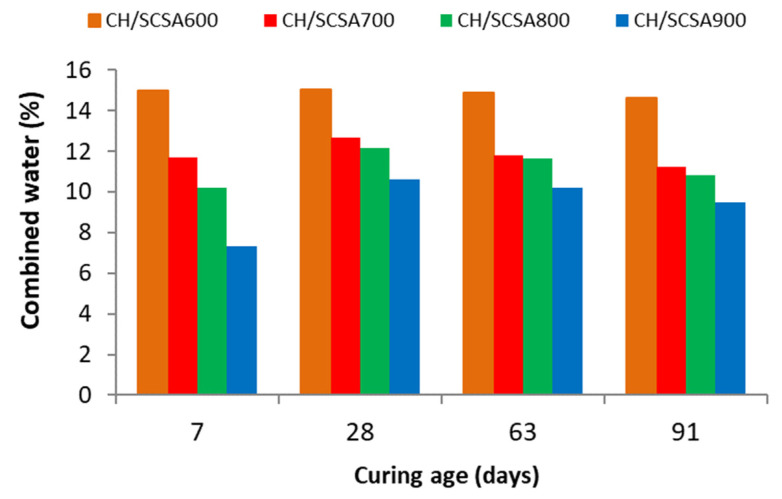
Combined water in the CH/SCSA pastes at different curing ages.

Combined water is a very useful parameter in TG because it is associated with the percentage of hydration products (CSH, CASH and CAH). The determination of this parameter is based on the percentage of the total mass loss (from 35 °C to 600 °C) minus the percentage of water loss from 520 °C to 580 °C. The percentage of combined water in the products formed in the pastes are shown in Figure 6.

These results confirm what has been discussed because, as age increases, larger amounts of hydration products form, which correlate with a higher combined water percentage. The CH/SCSA600 paste obtained the highest combined water percentages for all the curing ages because the values indicated high CH consumption. The CH/SCSA900 paste had the lowest combined water values of all the tested mixtures and for all ages.

**Figure 7:**
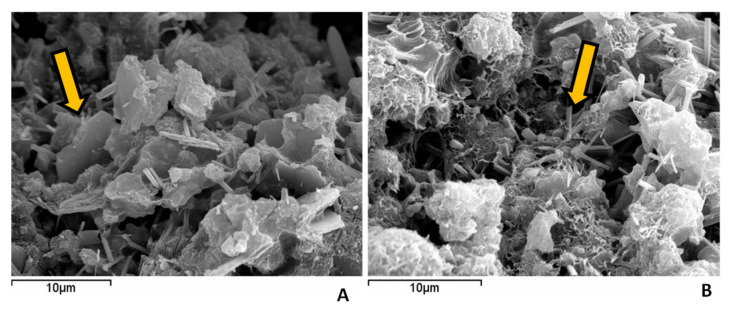
Scanning electron microscopy micrographs for the fractured surface of the 91-day-old CH/SCSA700 paste: (**A**) the arrow indicates the calcium aluminate hydrate plates; (**B**) the arrow denotes the presence of CSH needles.

Figure 7 depicts the SEM micrographs for the CH/SCSA700 paste at the age of 91 curing days. It also shows the formation of hydration products. The CSH-acicular (needles) shape is observed in the paste as described by other authors in similar CH/pozzolan systems and CASH plates [51]. This result corroborates the TG analysis for the CH/SCSA700 paste, with the formation of hydrated calcium aluminates and silicates and, therefore, calcium hydroxide consumption was high. It was very difficult to find CH particles because of the high lime fixation.

It is important to consider from the TG results of the blended cement pastes that the percentage of ash replacement for cement was 20%. In the CH pastes, there was more ash available (50%). Analyses were conducted on the control paste (100% cement) and the blended cement pastes (80% OPC, 20% ashes). Table 4 shows the fixed lime percentages (CH_cp_) in the blended cement pastes. In this study, it must be taken into account that there were two processes: a) the hydration of the cement components; b) the pozzolanic reaction of SCSA with the portlandite produced during the hydration of C*_3_*S and C*_2_*S of cement.

The reactivity of the ash in the blended cement pastes differed from that in the pastes with CH, with lower fixed lime values. However, in the pastes with SCSA600 and SCSA700, the prevalence of fixed lime was maintained in relation to other pastes, mainly the CEM/SCSA700 paste, which had the highest fixed lime values at 63 and 91 days. The irregularity of reactivity due to the age of most pastes can be attributed to the OPC system, which is more complex compared to the pastes obtained by CH. Frías and Cabrera [52] who found that the presence of ions (alkalis, chlorides, SO_4_^−2^, CO_3_^−2^) can modify the kinetics and development of hydrated phases. Thus, the fixed lime of the blended cement pastes was at least 25% lower than in those pastes with CH at 91 curing days. This difference has also been found by Frías and Cabrera [52]. According to these researchers, this fact is the consequence of the way in which the CH in cement paste is available and how it is slowly released to the hydration of cement, which may react later with the pozzolan. This fact is different from what occurs in pastes with hydrated lime, in which CH is completely available during early periods and then reacts with pozzolan. The comparison of the lime fixation results to those obtained by other authors showed that lime fixation was higher with the ashes calcined at 600 and 700 °C. Moraes et al. [23] obtained lime fixation values of around 38% in pastes with 15% SCSA at 28 curing days. Payá et al. [39] reported lime fixation values of around 43% in pastes with 30% SCBA at 56 curing days.

**Figure 8:**
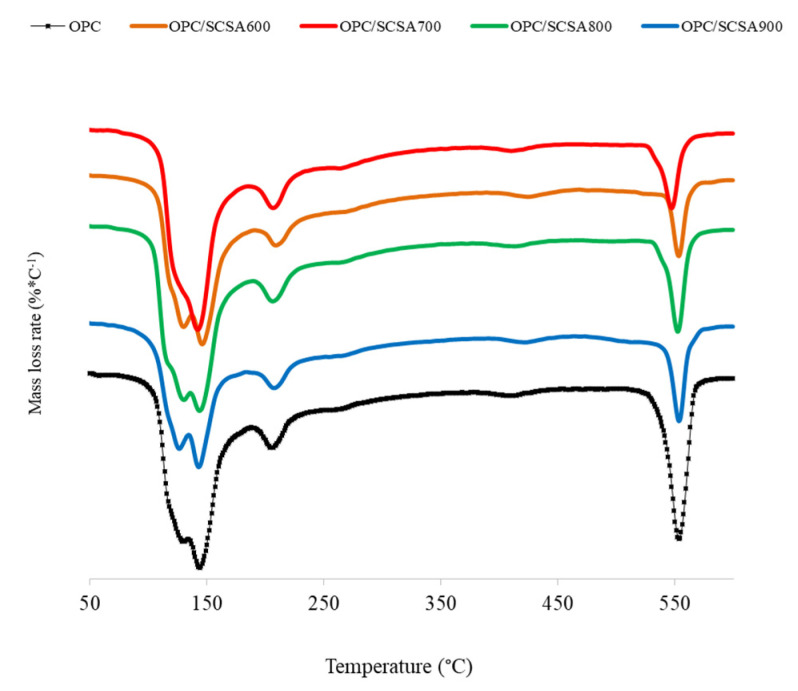
DTG curves for the blended cement pastes at 28 curing days.

The DTG curves of the blended and control pastes at 28 curing days are shown in Figure 8. On these curves, three zones (1, 2 and 3) related to the dehydration of ettringite and CSH (between 100 °C and 180 °C—zone 1), CASH and CAH (from 180 °C to 250 °C—zone 2) and CH dehydroxylation (from 520 °C to 580 °C—zone 3) were also identified [34].

### 3.4. Compressive Strength of Pastes

The mechanical behavior of pastes at the ages of 28 and 91 days was investigated by compressive strength testing. A statistical analysis was used with compressive strength as the dependent variable and the evaluated factor was paste type (blended and control).

**Figure 9:**
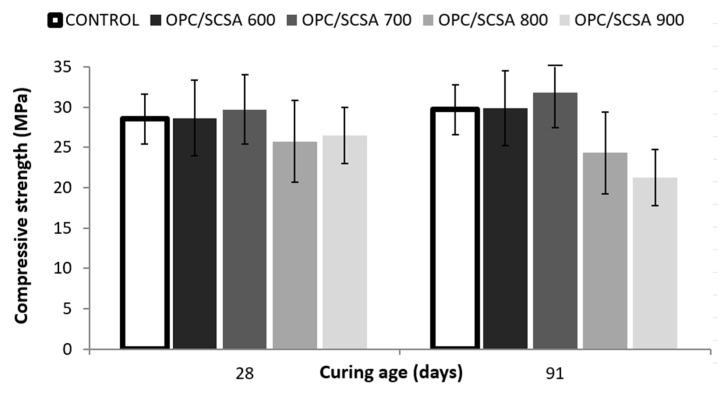
Compressive strength of pastes at 28 and 91 curing days.

Of all the paste types, when considering both curing ages, the CEM/SCSA600 and CEM/SCSA700 pastes demonstrated the best mechanical performance and did not present a statistically significant difference compared to the control paste (Figure 9). These results are consistent with those obtained by TG, where the sugarcane straw calcined at 600 °C and 700 °C presented greater lime fixation.

Based on the compressive strength results, there are marked indications that cementitious matrices with the 20% cement replacement, especially when using ashes SCSA600 or SCSA700, could be used without harming mechanical behavior. 

These results were compared to those of other authors who have used ashes from sugarcane bagasse waste. It should be noted that they had mortars, and not pastes, in most cases. Moraes et al. [23] applied SCSA as a cement substitute at 15%, 20%, 25% and 30%, They studied compressive strength evolution from 3 to 90 curing days. They concluded that the mortars with 20% SCSA always obtained similar compressive strength values to those of the control mortar for all curing ages; for example, at 28 days, the control mortar obtained 41.56 MPa versus the mortar with 20% SCSA, which obtained 42.82 MPa. De Siqueira and Cordeiro [53] prepared mixtures with 81% clinker, 14% SCBA and 5% gypsum. The mortars cured for 84 days obtained a compressive strength value of 62.1 MPa versus the control mortar with 54.7 MPa. Barbosa and Cordeiro [54] explored the use of three different SCBAs calcined at 600 °C to substitute 20% OPC. Those authors reported that the mortar with SCBA yielded 75.9 MPa of compressive strength at 28 curing days versus 64.5 MPa for the control mortar. 

The results obtained in the bibliography for mortars that employ bagasse waste are usually similar to or higher than the corresponding control system in all cases. This behavior demonstrates the positive contribution of this ash type when preparing cementing systems. 

### 3.5. Porosimetry of Pastes by the MIP Technique

**Figure 10:**
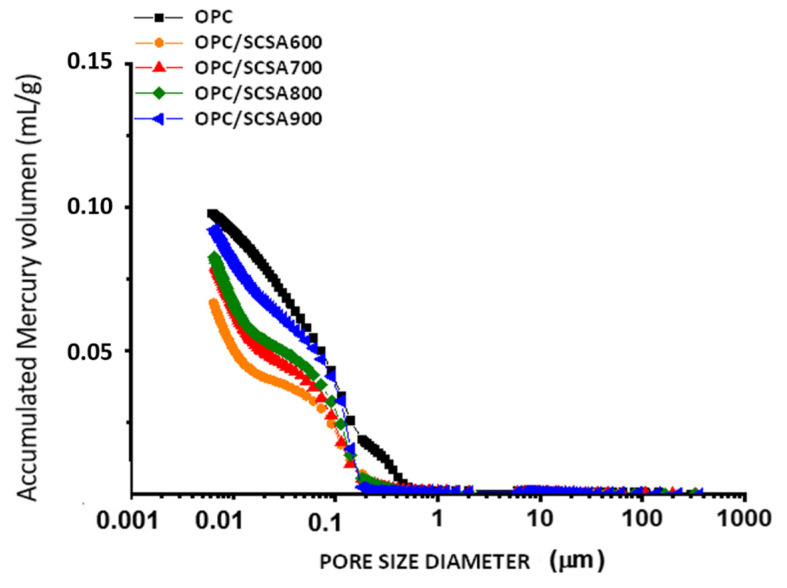
Mercury intrusion porosimetry results for the pastes cured for 91 days: accumulated curve.

**Figure 11:**
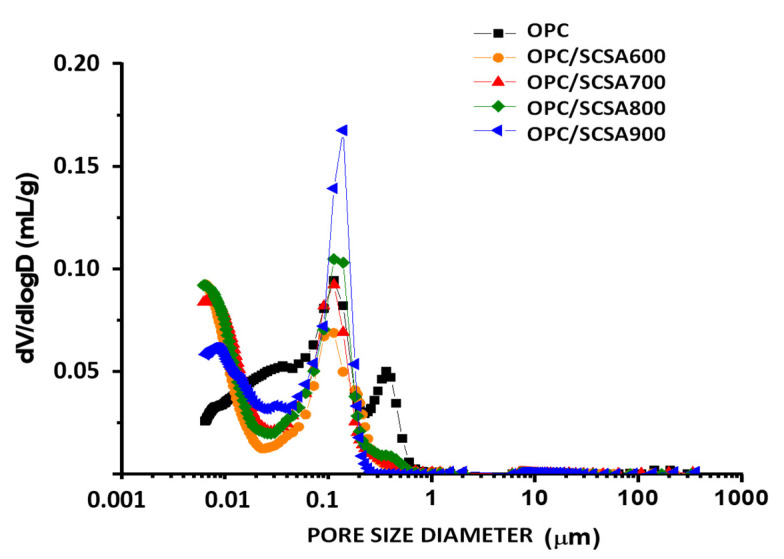
Pore size distribution of the pastes cured for 91 days.

Figure 10 shows the accumulated mercury volume curves for the equivalent diameters of the control paste and the ash-containing pastes after 91 curing days. Figure 11 depicts the curves with the discrete pore size distribution for these pastes.

It can be generally verified that the microstructures of all the pastes contained mainly gel pores (<0.05 µm) and small capillary pores (between 0.05 µm and 1 µm). Paste CEM/SCSA600 had the smallest total pore volume (0.068 mL·g^−1^). This value was significantly lower than that found for the control paste (0.098 mL·g^−1^). This means that the pozzolanic reaction offers benefits for the microstructure of the cured paste. 

The control paste had the largest volume of capillary pores, as seen in Figure 11. The ash-containing pastes showed a higher densification of the microstructure. Therefore, at 91 curing days, porous structure refinement was observed in the pastes with ash in relation to the structure of the control paste, with the best behavior noted for SCSA600.

Both pastes CEM/SCSA800 and CEM/SCSA900 had a larger total pore volume than pastes CEM/SCBA600 and CEM/SCBA700, which corroborates the results of the simple compression test, in which these pastes showed worse mechanical performance. The distribution of gel pores may be related to the CSH formation in these pastes, which consequently improves the material’s mechanical properties despite the lower OPC content (20% less). The greater the CSH formation, the more dense and less susceptible the material’s structure is to fracture [55]. Thus, the presented results corroborate the compressive strength results presented for pastes CEM/SCSA600 and CEM/SCSA700. 

## 4. Conclusions

Sugarcane straw was calcined at 600, 700, 800 and 900 °C. The prepared ashes had 61–65.7% SiO_2_, and the sum of acid oxides (silicon, aluminum and iron) fell within the 77.2–79.8% range. The chemical composition of the SCSA samples met the conditions stipulated by standards and/or practical applications for mineral additions. The XRD analysis showed that ashes presented crystalline phases but also had an amorphous phase, according to the halo found in the diffractogram Bragg angle, 2θ between 20° and 30°, which indicates their potential pozzolanic reactivity. The particle size analysis indicated that 90% of particles had equivalent diameters < 45 µm; SCSA600 and SCSA700 presented a large specific surface, which denotes significant internal porosity.

Regarding the reactivity of ahes, the calorimetric analysis demonstrated that ashes produced a delayed hydration process. The paste evaluation by TG revealed that all the ashes developed a high fixed lime percentage. Specifically, SCSA600 and SCSA700 showed the greatest reactivity, with ~85% for the hydrated lime pastes (50CH/50SCSA) and ~60% for the cement pastes (20% replacement). 

Regarding the contribution of ashes to the mechanical properties of pastes and microstructure, systems with ashes SCSA600 and SCSA700 (20% replacement) displayed similar mechanical behavior to that of the control paste, even at the early age of 7 days, which confirms the pozzolanic contribution to compressive strength. For those pastes, the mercury intrusion porosimetry test indicated optimal gel and capillary pore distribution, with a reduction in pore size and total porosity versus the control paste. 

As a general conclusion, sugarcane straw calcination at 600 °C and 700 °C yielded excellent biomass-derived pozzolans. Their reactivity (lime fixation) promoted good compressive strength development and an appropriate microstructure in the 20% OPC replacement systems.

In the future, the characteristics of sugarcane straw ashes generated in a pilot plant should be studied (more real/practical conditions), and, of course, properties in fresh mortars and concretes (workability, setting, rheology, etc.), and in the hardened state (compressive and bending strengths, shrinkage, porosity, elastic modulus, etc.) should be evaluated.

## Figures and Tables

**Table 1:** Pastes produced for the experiments.

Samples (Pastes)	Binder	Ash	Ash:Binder	w/b Ratio
CH/SCSA600	CH	SCSA600	50:50	1.0
CH/SCSA700	CH	SCSA700		
CH/SCSA800	CH	SCSA800		
CH/SCSA900	CH	SCSA900		
Control	OPC	-	-	0.5
CEM/SCSA600	OPC	SCSA600	20:80	
CEM/SCSA700	OPC	SCSA700		
CEM/SCSA800	OPC	SCSA800		
CEM/SCSA900	OPC	SCSA900		

**Table 2:** Chemical composition (mass/%) of ashes.

Ashes	SiO_2_	Al_2_O_3_	K_2_O	Fe_2_O_3_	CaO	SO_3_	MgO	P_2_O_5_	TiO_2_	Cl	MnO	LOI
SCSA600	64.4	9.3	5.4	3.9	3.8	3.3	2.5	2.0	1.2	0.7	0.2	2.9
SCSA700	61.0	9.2	6.9	5.0	4.4	3.9	2.8	2.3	1.3	0.6	0.3	2.1
SCSA800	62.3	8.6	6.4	4.6	5.2	4.3	2.7	2.2	1.1	0.2	0.4	1.7
SCSA900	65.7	9.6	5.4	4.5	4.1	2.6	2.8	2.5	1.2	0.1	0.2	0.9

**Table 3:** Fixed lime (CH_hlp_, %) of the CH pastes for different curing ages.

Pastes	Curing Age (Days)			
	7	28	63	91
	Fixed Lime CH_hlp_ (%)			
CH/SCSA600	88.07	90.01	91.71	87.23
CH/SCSA700	76.14	79.66	86.35	83.24
CH/SCSA800	72.37	78.31	80.56	79.40
CH/SCSA900	50.71	65.35	72.90	74.15

**Table 4:** Fixed lime (CH_cp_, %) of the blended cement pastes for different curing ages.

Pastes	Curing Age (Days)				
		7	28	63	91
	Fixed Lime CH_cp_ (%)				
CEM/SCSA600		40.75	53.39	51.56	57.66
CEM/SCSA700		45.46	51.37	57.12	67.89
CEM/SCSA800		35.42	35.31	41.25	40.62
CEM/SCSA900		31.14	29.59	31.43	33.55

## Data Availability

Data will be made available on request.

## References

[1] Georgiades M., Shah I.H., Steubing B., Cheeseman C., Myers R.J. (2023). Prospective life cycle assessment of European cement production. Conserv. Recycl..

[2] Schneider M. (2019). The cement industry on the way to a low-carbon future. Cem. Con. Res..

[3] Babu S., Rathore S.S., Singh R., Kumar S., Singh V.K., Yadav S.K., Yadav V., Raj R., Yadav D., Shekhawat K. (2022). Exploring agricultural waste biomass for energy, food and feed production and pollution mitigation: A review. Biores. Tech..

[4] Zhai J., Burke I.T., Stewart D.I. (2021). Beneficial management of biomass combustion ashes. Renew. Sust. Ener. Rev..

[5] Jittin V., Rithuparna R., Bahurudeen A., Pachiappan B. (2021). Synergistic use of typical agricultural and industrial by-products for ternary cement: A pathway for locally available resource utilization. J. Clean. Prod..

[6] Charitha V., Athira V.S., Jittin V., Bahurudeen A., Nanthagopalan P. (2021). Use of different agro-waste ashes in concrete for effective upcycling of locally available resources. Constr. Build. Mater..

[7] Ramos T., Matos A.M., Sousa-Coutinho J. (2013). Mortar with wood waste ash: Mechanical strength carbonation resistance and ASR expansion. Constr. Build. Mater..

[8] Zhang Z., Yang F., Liu J.C., Wang S. (2020). Eco-friendly high strength, high ductility engineered cementitious composites (ECC) with substitution of fly ash by rice husk ash. Cem. Concr. Res..

[9] Hasan N.M.S., Sobuz M.H.R., Khan M.M.H., Mim N.J., Meraz M.M., Datta S.D., Rana M.J., Saha A., Akid A.S.M., Mehedi M.T. (2022). Integration of Rice Husk Ash as Supplementary Cementitious Material in the Production of Sustainable High-Strength Concrete. Materials.

[10] Endale S.A., Taffese W.Z., Vo D.H., Yehualaw M.D. (2023). Rice Husk Ash in Concrete. Sustainability.

[11] El-Nadoury W.W. (2022). Eco-friendly concrete using by-products as partial replacement of cement. Front. Mater..

[12] Xu W., Lo T.Y., Memon S.A. (2012). Microstructure and reactivity of rich husk ash. Cons. Build. Mat..

[13] União da Indústria de Cana-de-Açúcar e Bioenergia “Sobre a Unica”. https://unica.com.br/sobre-a-unica/.

[14] Gopinath A., Bahurudeen A., Appari S., Nanthagopalan P. (2018). A circular framework for the valorisation of sugar industry wastes: Review on the industrial symbiosis between sugar, construction and energy industries. J. Clean. Prod..

[15] Chi M.C. (2012). Effects of sugar cane bagasse ash as a cement replacement on properties of mortars. Sci. Eng. Comp. Mat..

[16] Larissa L.C., Marcos M.A., Maria M.V., de Souza N.S.L., de Farias E.C. (2020). Effect of high temperatures on self-compacting concrete with high levels of sugarcane bagasse ash and metakaolin. Constr. Build. Mater..

[17] França S., Sousa L.N., Saraiva S.L.C., Ferreira M.C.N.F., Silva M.V.d.M.S., Gomes R.C., Rodrigues C.d.S., Aguilar M.T.P., Bezerra A.C.d.S. (2023). Feasibility of Using Sugar Cane Bagasse Ash in Partial Replacement of Portland Cement Clinker. Buildings.

[18] Ribeiro D.V., Morelli M.R. (2014). Effect of calcination temperature on the pozzolanic activity of Brazilian sugar cane bagasse ash (SCBA). Mater. Res..

[19] Bahurudeen A., Kanraj D., Gokul Dev V.V., Santhanam M. (2015). Performance evaluation of sugarcane bagasse ash blended cement in concrete. Cem. Concr. Compos..

[20] Villar-Cociña E., Valencia-Morales E., González-Rodríguez R., Hernández-Ruíz J. (2003). Kinetics of the pozzolanic reaction between lime and sugar cane straw ash by electrical conductivity measurement: A kinetic–diffusive model. Cem. Concr. Res..

[21] Guzmán A., Gutiérrez C., Amigó V., de Gutiérrez M.R., Delvasto S. (2011). Valoración puzolánica de la hoja de la caña de azúcar. Mater. Construct..

[22] Villar-Cociña E., Frías M., Savastano H., Rodier L., Sánchez de Rojas M.I., Sáez del Bosque I.F., Medina C. (2021). Quantitative Comparison of Binary Mix of Agro-Industrial Pozzolanic Additions for Elaborating Ternary Cements: Kinetic Parameters. Materials.

[23] Moraes J.C.B., Akasaki J.L., Melges J.L.P., Monzó J., Borrachero M.V., Soriano L., Payá J., Tashima M.M. (2015). Assessment of sugar cane straw ash (SCSA) as pozzolanic material in blended Portland cement: Microstructural characterization of pastes and mechanical strength of mortars. Constr. Build. Mater..

[24] Pane I., Hansen W. (2005). Investigation of blended cement hydration by isothermal calorimetry and thermal analysis. Cem. Concr. Res..

[25] Wilińska I., Pacewska B., Antonovič V. (2022). Hydration Processes of Four-Component Binders Containing a Low Amount of Cement. Materials.

[26] Zhang Y., Sun W., Liu S. (2002). Study on the hydration heat of binder paste in high-performance concrete. Cem. Concr. Res..

[27] Bakolas A., Aggelakopoulou E. (2019). Pozzolanic activity of natural pozzolan–lime pastes and physicomechanical characteristics. J. Therm. Anal. Calorim..

[28] Moropoulou A., Bakolas A., Aggelakopoulou E. (2004). Evaluation of pozzolanic activity of natural and artificial pozzolans by thermal analysis. Therm. Acta.

[29] Cordeiro G.C., Toledo Filho R.D., Tavares L.M., Fairbairn E.M.R. (2008). Pozzolanic activity and filler effect of sugar cane bagasse ash in Portland cement and lime mortars. Cem. Concr. Compos..

[30] (2018). Portland Cement Requirements.

[31] Natalli J.F., Thomaz E.C.S., Mendes J.C., Peixoto R.A.F. (2021). A review on the evolution of Portland cement and chemical admixtures in Brazil. Rev. IBRACON Estrut. Mater..

[32] (2022). Standard Test Method for Chemical Analysis of Hydraulic Cement.

[33] (2011). Cement—Part 1: Composition, Specifications and Conformity Criteria for Common Cements.

[34] McCarter W.J., Tran D. (1996). Monitoring pozzolanic activity by direct activation with calcium hydroxide. Cons. Build. Mat..

[35] Singh N.B., Singh V.D., Rai S. (2000). Hydration of bagasse ash-blended Portland cement. Cem. Concr. Res..

[36] Ganesan K., Rajagopal K., Thangavel K. (2007). Evaluation of bagasse ash as supplementary cementitious material. Cem. Concr. Compos..

[37] Sata V., Tangpagasit J., Jaturapitakkul C., Chindaprasirt P. (2012). Effect of W/B ratios on pozzolanic reaction of biomass ashes in Portland cement matrix. Cem Concr. Compos..

[38] (2019). Portland Cement—Determination of Compressive Strength.

[39] Payá J., Monzó J., Borrachero M.V., Díaz-Pinzón L., Ordónez L.M. (2002). Sugar-cane bagasse ash (SCBA): Studies on its properties for reusing in concrete production. J. Chem. Tech. Biotech..

[40] Siqueira A.A., Cordeiro G.C. (2022). Properties of binary and ternary mixes of cement, sugarcane bagasse ash and limestone. Constr. Build. Mater..

[41] (2023). Standard Specification for Coal Ash and Raw or Calcined Natural Pozzolan for Use in Concrete.

[42] Frías M., Villar-Cociña E., Valencia-Morales E. (2007). Characterisation of sugar cane straw waste as pozzolanic material for construction: Calcining temperature and kinetic parameters. Waste Manag..

[43] Chandrasekhar S., Pramada P.N., Majeed J. (2006). Effect of calcination temperature and heating rate on the optical properties and reactivity of rice husk ash. J. Mater. Sci..

[44] Cordeiro G.C., Sales C.P. (2016). Influence of calcining temperature on the pozzolanic characteristics of elephant grass ash. Cem. Concr. Compos..

[45] Xiong L., Sekiya E.H., Sujaridworakun P., Wada S., Saito K. (2009). Burning Temperature Dependence of Rice Husk Ashes in Structure and Property. J. Metals Mat. Min..

[46] Hay R., Li L., and Celik K. (2022). Shrinkage, hydration, and strength development of limestone calcined clay cement (LC3) with different sulfation levels. Cem. Concr. Compos..

[47] Zunino F., Scrivener K. (2019). The influence of the filler effect on the sulfate requirement of blended cements. Cem Concr. Res..

[48] Neto J.S.A., De la Torre A.G., Kirchheim A.P. (2021). Effects of sulfates on the hydration of Portland cement—A review. Constr. Build. Mater..

[49] Krishnan S., Emmanuel A.C., Bishnoi S. (2019). Hydration and phase assemblage of ternary cements with calcined clay and limestone. Constr. Build. Mater..

[50] Ruviaro A.S., dos Santos Lima G.T., Silvestro L., Barraza M.T., Rocha J.C., de Brito J., Gleize P.J.P., Pelisser F. (2023). Characterization and investigation of the use of oat husk ash as supplementary cementitious material as partial replacement of Portland cement: Analysis of fresh and hardened properties and environmental assessment. Cons. Build. Mat..

[51] De Souza L.M.S., Fairbairn E.D.M.R., Toledo Filho R.D., a Cordeiro G.C. (2014). Influence of initial CaO/SiO_2_ ratio on the hydration of rice husk ash-Ca(OH)_2_ and sugar cane bagasse ash-Ca(OH)_2_ pastes. Quim. Nova.

[52] Frías M., Cabrera J. (2001). Influence of MK on the reaction kinetics in MK/lime and MK-blended cement systems at 20 °C. Cem. Concr. Res..

[53] Siqueira A.A., Cordeiro G.C. (2022). Sustainable Cements Containing Sugarcane Bagasse Ash and Limestone: Effects on Compressive Strength and Acid Attack of Mortar. Sustainability.

[54] Barbosa F.L., Cordeiro G.C. (2021). Partial cement replacement by different sugar cane bagasse ashes: Hydration-related properties, compressive strength and autogenous shrinkage. Constr. Build. Mater..

[55] Martirena J.F., Middendorf B., Gehrke M., Budelmann H. (1998). Use of wastes of the sugar industry as pozzolana in lime-pozzolana binders: Study of the reaction. Cem. Concr. Res..

